# Assessing the surge capacity of hospitals in Ugandan health care systems in managing the COVID-19 pandemic

**DOI:** 10.1192/j.eurpsy.2023.1658

**Published:** 2023-07-19

**Authors:** D. Jephthah, I. Ddumba

**Affiliations:** 1 Nursing, Victoria University; 2 research AND innovation, AFRICAN RESEARCH CENTER 4 AGEING & DEMENTIA, Kampala, Uganda

## Abstract

**Introduction:**

The increasing cases of COVID-19 poses a threat on the over strained health care systems, especially in developing countries. Health care systems might need a surge to accommodate the ever increasing number of COVID1-19 cases. Hence, we assessed the Ugandan healthcare systems’ capacity to accommodate the surge in the increased caseloads, which might need admission and critical care due to COVID-19.

**Objectives:**

Understanding the health systems capacity to accomadate the surge in increase caseload of COVID-19

**Methods:**

We assumed that 2% of the Uganda population get symptomatic infections by COVID-19 based on modelled estimates of Uganda and ascertained the healthcare systems surge capacity for COVID-19 under three transmission curves scenarios; 6, 12 and 18 months. We estimated four measures for hospital surge capacity; ICU bed surge capacity, ICU bed tipping point, hospital bed capacity and hospital bed tipping point. Estimates were made for national level and 132 district local government.

**Results:**

The capacity of Ugandan health care system to accommodate the increasing numbers of cases due to COVID-19 is hindered by the lack of oxygen. Only 9 in 20 (46%) of hospital beds had oxygen supply. The hospital bed surge capacity varied across districts. Under the 12 months transmission scenario, the proportion of hospital with available beds, that would accommodate COVID-19 cases varied from 4% in Karomoja district, to 84% in Kampala district. The Ugandan healthcare systems faces a critical gap in ICU beds and ventilator capacity. Only 48 out of 132 districts had at least 1 ICU unit. An additional 2,247 bed and 2,756 ventilators (12 months transmission curve) will be needed to accommodate the caseloads due to COVID-19.

**Conclusions:**

The capacity for Ugandan healthcare systems to manage to manage the COVID-19 caseloads is minimal. There need to address the sub-national variations in bed surge capacity, ICU units and ventilators within the Ugandan healthcare system.

**Disclosure of Interest:**

None Declared

